# Immune-Modulating Lipid Nanomaterials for the Delivery of Biopharmaceuticals

**DOI:** 10.3390/pharmaceutics15061760

**Published:** 2023-06-18

**Authors:** Songhee Kim, Boseung Choi, Yoojin Kim, Gayong Shim

**Affiliations:** School of Systems Biomedical Science and Integrative Institute of Basic Sciences, Soongsil University, Seoul 06978, Republic of Korea; sh221511@gmail.com (S.K.); cqhtmd@gmail.com (B.C.); kimyoojin120@gmail.com (Y.K.)

**Keywords:** lipid nanoparticles, biopharmaceutical delivery, immune modulation, RNA delivery, immunotherapy

## Abstract

In recent years, with the approval of preventative vaccines for pandemics, lipid nanoparticles have become a prominent RNA delivery vehicle. The lack of long-lasting effects of non-viral vectors is an advantage for infectious disease vaccines. With the introduction of microfluidic processes that facilitate the encapsulation of nucleic acid cargo, lipid nanoparticles are being studied as delivery vehicles for various RNA-based biopharmaceuticals. In particular, using microfluidic chip-based fabrication processes, nucleic acids such as RNA and proteins can be effectively incorporated into lipid nanoparticles and utilized as delivery vehicles for various biopharmaceuticals. Due to the successful development of mRNA therapies, lipid nanoparticles have emerged as a promising approach for the delivery of biopharmaceuticals. Biopharmaceuticals of various types (DNA, mRNA, short RNA, proteins) possess expression mechanisms that are suitable for manufacturing personalized cancer vaccines, while also requiring formulation with lipid nanoparticles. In this review, we describe the basic design of lipid nanoparticles, the types of biopharmaceuticals used as carriers, and the microfluidic processes involved. We then present research cases focusing on lipid-nanoparticle-based immune modulation and discuss the current status of commercially available lipid nanoparticles, as well as future prospects for the development of lipid nanoparticles for immune regulation purposes.

## 1. Introduction

**Figure 1:**
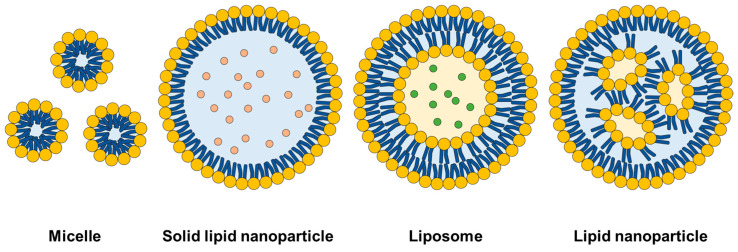
Types of lipid-based nanoparticles.

In the field of nanomedicine, lipid-based nanoparticles have garnered significant attention due to their distinctive properties and their potential for various applications in drug delivery [1,2]. Researchers have developed multiple types of lipid-based nanoparticles, each offering unique advantages and properties (Figure 1). Micelles, on the other hand, are self-assemblies of lipid monolayers in aqueous solutions, forming structures with a hydrophobic core [3]. The hydrophobic core of a micelle is particularly well-suited to the encapsulation of small hydrophobic molecules. They can serve as effective carriers for the delivery of hydrophobic drugs, enhancing solubility and stability. Solid lipid nanoparticles feature a surfactant shell encasing a solid lipid core matrix [4,5]. The solid matrix of solid lipid nanoparticles imparts stability and enables the controlled release properties, making them valuable in various drug delivery applications. Another notable lipid-based nanoparticle variant is liposomes, which consist of one or more lipid bilayers encompassing an aqueous core [6,7]. Liposomes can be categorized based on their lamellarity and size. Due to their unique structure, liposomes have the capacity to encapsulate both hydrophobic and hydrophilic small molecules, positioning them as versatile carriers for a wide array of therapeutic agents. Lipid nanoparticles (LNPs) represent a versatile class of lipid-based nanoparticles characterized by a lipid shell enveloping an inner core composed of reverse micelles [8,9,10]. This structure enables LNPs to effectively encapsulate and deliver diverse oligonucleotides, including siRNA, mRNA, and plasmid DNA. LNPs are renowned for their excellent stability and efficient intracellular delivery, making them highly suitable for nucleic-acid-based therapy.

Lipid-based nanoparticles offer a diverse range of options for drug delivery. Each type exhibits unique properties that can be tailored to meet the specific requirements of the therapeutic cargo. Factors such as the nature of the cargo, desired release kinetics, and the targeted delivery site play crucial roles in selecting the appropriate lipid-based nanoparticles. Ongoing research and development in this field hold immense promise for advancing drug delivery strategies and improving therapeutic outcomes.

Recently, LNPs have been actively investigated for the delivery of RNA-based biopharmaceuticals [11,12]. Although non-viral vectors such as LNP have lower efficiency than viral vectors, the former are biosafe and have the advantage of being able to carry large mRNA sequences [13,14]. Furthermore, LNPs can easily fulfill three requirements for nucleic acid delivery vehicles: efficient encapsulation to resist degradation by nucleases, a mechanism for targeted delivery and intracellular entry, and a manufacturing process that allows for the mass production of uniformly sized nanoparticles [15]. The cargo content and physicochemical properties (particle size, shape, surface charge, etc.) of LNPs can be controlled by adjusting the lipid composition [16].

Nanoparticles containing cationic lipids have been widely studied as carriers of genes and nucleic acids because of their ability to carry negatively charged nucleic acids and attach to the lipid bilayer of cells [17]. However, cytotoxicity due to cationic lipids and rapid elimination from the body are limitations [18]. To compensate for this, ionizable lipids that are neutral in circulation and positively charged only in endosomes have been developed and actively studied [19].

The use of microfluidic mixing enables the fabrication of LNPs that effectively shield biopharmaceuticals [20]. Conventional batch-mode mixing methods rely on the homogenization of the particle size through sonication and extrusion [21]. However, mass production using these methods is difficult, and high batch-to-batch variability is a drawback. In particular, low yields and complex processes have made the clinical transition of LNPs difficult. In contrast, microfluidic production techniques have the advantage of producing homogeneously sized nanoparticles via a simple process, and large quantities of these nanoparticles can be easily produced [20]. The size of the nanoparticles can also be effectively controlled using microfluidic devices [22]. siRNA-loaded LNPs smaller than 50 nm that are capable of tumor penetration have been prepared using this technique [20].

The first approved siRNA-loaded LNPs (Onpattro) demonstrated the potential of LNP production using a microfluidic-based ethanol injection method for medicinal applications [22]. The mRNA vaccines recently launched by Moderna and Pfizer are lipid nanoparticle formulations produced using microfluidic processes. In vaccine development, RNA, unlike proteins, has the advantage of being able to produce antigens in rapid response to mutations because the manufacturing process can be carried out quickly; however, the application of RNA in the body in a free form is difficult. With the commercialization of LNPs that deliver RNA, researchers are working intensively to develop lipid-based therapeutics that can be applied to the immune system. The personalized mRNA cancer vaccine (mRNA-4157/V940) based on neoantigen sequences developed by Moderna and MSD demonstrated remarkable phase II trial results, showing a 44% reduction in the risk of recurrence or death in melanoma when combined with checkpoint blockade therapy. Compared to conventional vaccine platforms, immunization with mRNA has the advantages of rapid translation of gene sequences, short action times, and rapid design and manufacturing [23]. For this reason, LNPs for RNA delivery have been widely used as vaccines against various infectious diseases and cancer.

The remarkable progress of mRNA cancer vaccines has led to a shift in focus towards the development of immunotherapeutic treatments using biopharmaceuticals [24]. Biopharmaceuticals offer a novel approach for leveraging the immune system’s potential in combating cancer. In this context, the choice of an appropriate delivery system plays a crucial role in achieving the effective and targeted delivery of biopharmaceutical agents. Among the available options, LNP delivery systems have garnered significant attention due to their versatility and proven efficacy in the field of mRNA vaccine development. In this review paper, we aim to examine the research on immunotherapy using LNP delivery systems, which serve as carriers for various biopharmaceutical cargos. Furthermore, we will discuss the development strategies for appropriate cancer vaccines based on different types of biopharmaceuticals.

## 2. Design of Lipid Nanoparticles

### 2.1. Typical Composition

Typical LNP formulations include phospholipids, cholesterol, positively charged glycolipids, and PEGylated lipids that make up the cell membrane [25]. Phospholipids, a component of cell membranes, act as a skeleton for LNPs and aid in the release of LNPs from endosomes [26]. Although considered as the backbone, their immunogenicity differs depending on the type of phospholipid; therefore, it is necessary to optimize the phospholipid composition [25]. Owing to its hydrophobic nature, cholesterol is placed between the lipid bilayers to increase the rigidity of the LNPs, thus increasing the stability of the latter. Polyethylene-glycol (PEG)-modified lipids provide a shielding layer that minimizes the non-specific binding of LNPs to proteins or cells in vivo [27]. However, immune responses of PEGylated nanoparticles have recently been reported, leading to research on alternative materials [27,28]. When PEGylated drugs are administered intravenously, the formation of anti-PEG antibodies may occur, leading to an Accelerated Blood Clearance (ABC) phenomenon with subsequent doses. Consequently, the drug can be rapidly eliminated from the bloodstream. These phenomena have been observed repeatedly in PEGylated drugs and nanoparticles, resulting in reduced therapeutic efficacy by lowering drug residence time in the bloodstream. In addition, the rate of elimination from the body differs based on whether the lipid tail structure is symmetrical versus asymmetrical [29].

### 2.2. Composition for Ionizable LNP

Positively charged lipids are classified as permanent or ionizable lipids. In the initial studies, cationic lipids were expected to form lipoplexes with negatively charged cargo for transport and to bind to anionic lipids in the body’s cell membranes to induce destabilization [18]. However, ionizable lipids have been used instead of permanent cationic lipids to avoid hemolysis and to enable rapid clearance from the circulation because of their positive charge [19].

The ionizable lipid structure consists of an acyl chain linked to a hydrophilic amine headgroup with an apparent pKa value of less than 7 [30,31]. Ionizable lipids are only positively charged at a low pH, e.g., when enclosing nucleic acids and inside endosomes, and have a near-neutral charge at a physiological pH [30]. In endosomes, positively charged LNPs promote fusion with the membrane and release nucleic acids into the cytoplasm [31]. As an advantage, ionizable lipids can fully encapsulate nucleic acids and increase the intracellular delivery rate while reducing toxicity.

## 3. Lipid Nanoparticles for Biopharmaceutical Delivery

The most widely studied formulations for siRNA delivery, both clinical and non-clinical, generally contain ionizable lipids, such as DLin-MC3-DMA. An siRNA medicine (Onpattro) using LNPs, containing the DLin-MC3-DMA lipid as a core component, was approved as the first siRNA therapy for the treatment of polyneuropathy in adult hereditary transthyretin-mediated (hATTR) amyloidosis [31]. Even if not intended for therapeutic purposes, siRNA-loaded LNPs are an important research tool in biotechnology [32]. They can partially replace the highly labor-intensive use of transgenic knockout mice by inhibiting target genes in the liver, lungs, and cardiac endothelial cells, which are the main target tissues for LNPs.

mRNA is a strongly negatively charged, highly water-soluble biopolymer that is difficult to introduce into the cell on its own. Rapid degradation by extracellular RNases is a major barrier to the use of mRNA as a therapeutic agent [33]. The modification of nucleic acid sequences and the use of appropriate delivery vehicles have played important roles in the successful development of mRNA therapeutics. The modification of uridine present in the mRNA sequence to create pseudouridine (Ψ) has been shown to improve the stability and translation efficiency of mRNA [23]. Currently, the most advanced non-viral vector serving as a carrier of (IVT) mRNA transcribed in vitro is LNP, and its delivery efficacy has been demonstrated in rodents and non-human primates [34]. The clinical use of IVT-mRNA-LNPs is being explored because they can rapidly express the desired protein without the risk of mutagenesis, which is a concern for viral vectors. IVT-mRNA-LNPs can also be designed and synthesized using a very simple process, as compared to the design, expression, and purification of traditional recombinant proteins [35].

A variety of design options exist for LNP-based CRISPR/Cas ribonucleoprotein delivery systems, depending on whether the Cas protein is to be delivered as DNA, mRNA, or ribonucleoprotein. In the microfluidic process, proteins and nucleic acids (gRNA) can be enclosed inside LNPs, a process which has the advantage of reducing the loss of DNA cleavage activity and reducing protein aggregation [36].

The efficacy of cationic/ionizable lipids may vary depending on the size of the nucleic acid, as well as the structure and degree of modification [37]. mRNA, which has a larger molecular size than siRNA, forms an inverted hexagonal nanostructure during the process of enclosure within the LNP [8]. Kauffman et al. optimized the composition of LNPs for mRNA delivery through the in vivo design-of-experiment (DoE) optimization of the composition of LNPs for siRNA delivery [26]. Therefore, modification of the delivery vehicle is required, even depending on the nucleic acid type, and LNPs optimized for one type of nucleic acid will not necessarily be effective for the delivery of other types of nucleic acids [37].

## 4. Target Organs of Lipid Nanoparticles

LNPs containing ionizable lipids have been studied for the systemic delivery of RNA therapeutics, but designing nanoparticles that can be delivered to target tissues beyond the liver remains challenging [38]. Most of the currently studied LNPs show affinity for the liver because it is a well-perfused organ that can take up intravenously injected cargo, and the slow blood flow and sinusoidal vasculature of the liver also play a role in aiding LNP distribution [39,40]. In addition, neutrally charged LNPs that enter the bloodstream bind to apolipoprotein E (ApoE), which is taken up through low-density lipoprotein receptors distributed in the liver [41]. Thus, neutral LNPs can rapidly accumulate in the liver without using ligands for molecules overexpressed by hepatocytes. Even if the LNP surface is PEGylated, it has been reported that PEGylated lipids and ApoE proteins are exchanged after injection into the bloodstream [42].

The challenge in expanding the use of LNPs is evolving them to have different tropisms in vivo [39]. Attempts have been made to modulate the surface charge of LNPs in order to deliver drugs to organs other than the liver. Lung-specific mRNA delivery has been achieved by increasing the proportion of persistent cationic lipids in LNPs; conversely, spleen-specific mRNA distribution has been observed when the surface negative charge is high [32].

The method of reducing the diameter of the nanoparticles for tumor penetration has also been studied. Using a microfluidic mixing technique, siRNA-LNPs with sizes of less than 50 nm were prepared and efficiently invaded the tumor microenvironment [20]. In another study, improved penetration into diseased tissues, such as tumors, was observed using LNPs with a diameter of 30 nm [18]. Recently, it was shown that LNPs as small as 20 nm in diameter can be produced by increasing the PEGylation ratio using microfluidic technology. These small LNPs are applicable not only to tumors but also to metastatic lymph nodes.

## 5. Immune-Modulating Lipid Nanoparticles

Various lipid-based nanoparticles have been studied for immunotherapeutic applications. mRNA delivery is expected to elicit antigen-specific immune responses through the addition of nanovaccine LNPs delivering siRNA, a method which has been investigated as a synergistic strategy for silencing the target mRNA to aid in immunotherapy. Antigenic peptides have also been delivered by enclosing them in LNPs, as summarized in Table 1.

### 5.1. siRNA-LNP

Antitumor immunotherapy was performed by delivering siRNA targeting heme oxygenase-1 as iLNPs [43]. Heme oxygenase-1 siRNA has been proposed as a novel immune checkpoint inhibitor that can induce the activation of antitumor T cells by inhibiting their expression by RNAi. The siRNA targeting heme oxygenase-1 was enclosed inside iLNPs containing a novel ionizable lipid via a microfluidic process. The surface of the iLNPs was modified with PD-L1 antibodies for selective uptake by myeloid and tumor cells. The antibody-loaded iLNPs induced heme oxygenase-1 silencing in tumor/myeloid cells, which led to different boosting effects. Heme oxygenase-1 silencing boosted Dox chemotherapy in the tumor cells and induced the polarization of macrophages in myeloid cells to activate immunotherapeutic activity. iLNPs inhibited melanoma primary tumor growth while inducing immunogenic cell death and inhibiting metastasis in the lung tissue.

Researchers developed a bio-reducible lipid that delivered siRNA to a mouse brain tumor to modulate the tumor microenvironment and treat glioblastoma multiforme (GBM) [46]. Although immunotherapies have achieved promising clinical results in the treatment of multiple cancers, GBM patients especially benefit from them because of the poor delivery rate across the blood–brain barrier (BBB). The pKa value of 9-O16B is approximately 6.5, and siRNA lipoplexes containing this lipid were able to cross the BBB via the endocytosis of cerebral vascular endothelial cells and the transport of siRNA to intracranial tumor tissues. The siRNA lipoplex delivered siRNA against CD47 and PD-L1 across the BBB into GBM in mice, resulting in the synergistic activation of a T-cell-dependent antitumor immune response in an orthotopic GBM model.

A study developed siRNA-LNPs with conformation-sensitive targeting ligands to treat inflammatory bowel disease [49]. To formulate the ligand, an anti-rat IgG2a monoclonal antibody (RG7 linker) was conjugated to the maleimide moiety exposed on the LNP surface. The affinity of the RG7 linker for binding to the mucosal vascular adhesion molecule-1 (MAdCAM-1)-Fc fusion protein resulted in the LNPs targeting α4β7 integrin. Integrin α4β7 is expressed in intestinal-homing leukocytes, and the LNPs developed in that study were specifically designed to recognize only the high-affinity conformation of integrin α4β7, which is specifically expressed on intestinal-homing leukocytes. Interferon-gamma-targeting siRNA was incorporated into LNPs via a microfluidic-based method and induced the interferon gamma silencing of inflammatory leukocytes by targeting HA α4β7, thereby restoring the balance of the intestinal immune response.

### 5.2. Gene-Editing LNP

Lipid–metal hybrid nanoparticles have been investigated as delivery systems for gene editing for reprogramming the tumor microenvironment [51]. Au metal clusters were entrapped in the aqueous phase of cationic LNPs via thin-film hydration and gold clustering. LNPs can deliver plasmid DNA encoding Cas9 protein and transforming growth factor-β (TGF-β) single-guide RNA. That study demonstrated that LNP-mediated TGF-β gene editing of the tumor microenvironment could reconstitute the tumor microenvironment, which is favorable for immune evasion. The TGF-β-gene-edited tumor microenvironment suppressed regulatory T cell differentiation and induced interferon gamma secretion. The photothermal treatment of TGF-β-gene-edited tumors ablated primary tumors and prevented distant tumors. In addition, the immunization of mice with LNPs effectively prevented the lung metastasis of melanoma cells.

### 5.3. mRNA-LNP

An mRNA-LNP that effectively induced antitumor immunity was prepared based on a design-of-experiment (DoE) method [45]. The lipid composition of the LNPs, which have a strong tendency to concentrate in the liver after systemic administration, was optimized for the purpose of the vaccine. A library of more than 30 LNPs was generated based on the hybrid design of Roquemore. Antibody-specific CD8 T cell responses were measured in the blood of mice systemically administered LNPs. Bayesian regression of the animal experimental results was used to identify novel lipid compositions for immunization. The compositions of the LNPs derived from the model were validated in mice, and the experimental data for LNPs containing DMG-PEG2000 and DSG-PEG2000 were found to be consistent with the predictions. The optimal LNPs loaded with E7 mRNA showed increased uptake by immune cells in the spleen, and the distribution of LNPs in the spleen was confirmed in non-primates and cynomolgus monkeys. The repeated administration of LNPs induced strong tumor-antigen-specific CD8 T cell responses in a syngeneic mouse TC-1 tumor model.

One study used modified mRNA-LNPs to immunize against mousepox, a fatal viral disease [23]. The researchers aimed to induce a strong immune response to the epitope TSYKFESV by enclosing the mRNA encoding the EVM158 gene of the *Ectromelia* virus in cationic LNPs. Interestingly, a comparison of the LNPs loaded with unmodified mRNA versus *N*(1)-methyl pseudouridine-modified mRNA showed that the unmodified mRNA induced adverse effects upon inoculation. In contrast, mice immunized with mRNA-LNPs containing pseudouridine did not develop mousepox through viral infection. A booster dose induced the activation of memory T cells and had a sustained effect.

### 5.4. Cyclic Di-Nucleotide-LNP

Harashima and colleagues developed cancer immunotherapy using an adjuvant delivery system based on immune status analysis in the tumor microenvironment [47]. The researchers defined the immune status parameter in the tumor microenvironment showing antitumor effects by analyzing gene expression in tumors that responded to the PD-1 antibody. They identified a 10-gene immune status panel (IS-panel-10) that affected the prognosis of various human cancers. Treatment with an agent that stimulates the interferon gene (STING) pathway with an LNP formulation (STING-LNP) showed remarkable antitumor efficacy within the range of gene expression for effective anti-cancer effects. YSK12-C4, a key component of STING-LNP, is a cationic lipid with high affinity for immune cells [52]. Following the strategy established through IS-panel-10, the combination of STING-LNP and the CTLA4 antibody in the 4T1 tumor model resulted in significant tumor suppression, despite the cancer being resistant to immunotherapy, including immune checkpoint inhibitors. These findings demonstrate the potential for analyzing the tumor microenvironment in developing cancer immunotherapies using LNP.

Liposomes loaded with STING agonists were designed to reverse the immune-suppressive tumor microenvironment of malignant pleural effusion (MPE) [48]. Liposomes containing phosphatidylserine were encapsulated with cyclic GMP-AMP, a STING agonist, in a complex with calcium phosphate. The phosphatidylserine on the outer layer of the liposomes enabled the delivery of the antigen-presenting cell-selective STING agonist. Calcium phosphate entrapped in cyclic GMP-AMP induced cytosolic delivery of the STING agonist via endosome pH-responsive release. PEGylated liposomes were more favorable for MPE retention and reducing unwanted extra-thoracic distribution compared to the non-PEGylated nanoparticles. STING-liposomes transported in MPE reprogrammed immunosuppressive myeloid cells into an inflammatory phenotype and activated the effector function of CD8 T cells and NK cells. Combination therapy with liposomes and PD-L1 antibody reduced the volume of MPE and increased the survival rate of the MPE mouse model.

### 5.5. Peptide-LNP

A nanovaccine in liposomal formulation was designed to treat Alzheimer’s disease without the side effects of immunotherapy [44]. Aβ1-42 peptides were incorporated into PEGylated liposomes with rapamycin to induce immune tolerance. Nanovaccine uptake by immature dendritic cells induced the production of anti-Aβ antibodies and Aβ-specific Treg cells. Liposomes without rapamycin, used as a control, could clear Aβ plaques through the induction of anti-Aβ antibodies but caused neuroinflammation due to Aβ-specific Th1 cells. In contrast, nanovaccines containing rapamycin induced the production of Aβ-presenting tolerogenic dendritic cells and Aβ-specific Tregs. The secretion of anti-inflammatory factors by these cells inhibited Th1 cell activation. Intramuscular injection of a nanovaccine suppressed cognitive impairment in a 5xFAD transgenic mouse model.

## 6. Challenges and Perspectives

To date, LNP products approved as biopharmaceutical delivery systems include a COVID-19 vaccine and treatment for inherited amyloidosis (Table 2). Prior to the launch of nucleic-acid-incorporated LNPs, FDA-approved lipid-based drugs utilized lipids to solubilize insoluble small molecules and mitigate side effects. Alnylam Pharmaceuticals has been focusing on developing lipids for siRNA delivery since the early 2000s and eventually developed the first siRNA medicine [53].

Following ONPATTRO, Alnylam Pharmaceuticals successfully launched a series of siRNA therapeutics, including GIVLAARI, OXLUMO, Leqvio (Norvatis), and AMVUTTRA. Notably, ONPATTRO’s subsequently produced siRNA drugs are all sugar–siRNA conjugates. This technology links three *N*-acetylgalactosamines (GalNAc) to siRNA molecules. GalNAc is a trivalent ligand that targets the asialoglycoprotein receptor that is abundantly expressed in hepatocytes. The eight products in the pipeline and currently in clinical trials are also GalNAc ligand–siRNA conjugates. One of the reasons why Alnylam Pharmaceuticals is no longer focusing on LNPs is their inefficiency. The mass of LNPs required to enclose the siRNA requires a very high content of excipients compared to the active substance (siRNA). In the case of ONPATTRO, infusions were performed every 3 weeks for 80 min and were accompanied by pretreatment with multiple anti-inflammatory drugs to minimize reactions to the nanoparticles. Because LNPs can activate the immune system and cause anaphylactic-like shock, the risk of an acute immune response must be recognized, and solutions must be prepared [54].

There are several challenges in the mass production of LNP formulations that are currently being studied for mRNA delivery. Depending on their type, ionizable lipids can be less economical than traditional cationic lipids, and there are less clinical data on the former [33]. Owing to their long half-life, MC3 lipids have the potential to cause adverse effects when used in chronic treatment [54]. MC3 is not completely metabolized within the body, leading to the presence of residual metabolites. This increases the potential for adverse effects resulting from the accumulation of metabolites when the LNP is used for chronic conditions requiring long-term administration. Extensive research data are required to demonstrate an acceptable safety profile. In the case of RNA encapsulated inside LNPs, the nature of the formulation may limit the stability of the RNA, often requiring immediate use upon manufacture [55].

Improving the uptake of LNPs by various cell types is also an area of active research for expanding the utilization potential of LNPs. Currently, LNP therapies approved in the United States are either systemically administered to target hepatocytes or locally administered to muscles [56]. To understand the distribution of LNPs in the body, it is necessary to consider the properties of the LNP surface that influence the composition of the protein corona. The protein corona not only interferes with ligands on the surface of the LNPs but also changes the surface properties and size of the LNPs. The interaction between the protein corona and LNPs can determine the colloidal stability of the LNPs [57]. Therefore, for LNPs developed for systemic administration, it is important to characterize the effect of the interaction with serum upon siRNA delivery [57].

Typically, the optimization of the design of LNPs is based on the evaluation of their physicochemical properties in vitro and their cellular uptake in cultured cells [58]. However, it is difficult to reconcile the results of these evaluations with the fate of LNPs after administration to the body, as it is difficult to predict their interaction with cells in the blood, the formation of a protein corona, and biological barriers. In addition, quantitative methods for determining in vivo tissue delivery are limited. It is difficult to determine the distribution of each lipid in LNPs using fluorescence imaging methods with fluorescently labeled nucleic acids. Because there are limits to the types of fluorescence that can be quantified in a single animal, it is necessary to devise a quantification method using granular labeling methods such as mass cytometry.

The development of cancer vaccines faces challenges in addressing the diverse array of tumor types and variants [59]. Immuno-oncology therapies necessitate a comprehensive understanding of individual tumor characteristics and unique antigens. An integral step in personalized therapy using neoantigens involves the identification and discovery of these specific antigens, as they are individual-specific and distinct from the commonly known oncogenes [60]. Neoantigens can be categorized as shared neoantigens or individual-specific neoantigens. Shared neoantigens are not specific to an individual or tumor type and serve as targets for off-the-shelf therapeutics, while individual-specific neoantigens are highly tailored to an individual’s tumor and are utilized for the development of personalized medicine.

Neoantigens hold significant potential for the development of various types of biopharmaceuticals, including mRNA, DNA, and peptides [61]. In this regard, the utilization of LNPs is believed to offer possibilities for expanding the repertoire of biopharmaceuticals. For instance, in the case of individual-specific neoantigens, customizing the production of neoantigen information obtained through the genome-wide sequencing of tumor tissue is crucial, making the mRNA formulations currently under development a suitable option. However, for shared neoantigens, alternative biopharmaceuticals may also be considered, as they have broader applicability across a wider range of patients.

By leveraging LNPs as effective delivery systems, the development of biopharmaceuticals targeting both shared and individual-specific neoantigens can be facilitated. LNPs offer advantages such as efficient encapsulation, enhanced stability, and targeted delivery, making them promising candidates for the delivery of diverse biopharmaceutical payloads. The potential synergy between LNPs and neoantigen-based therapies opens up new avenues for personalized and effective cancer immunotherapy, contributing to advancements in the field of precision medicine.

## 7. Conclusions

Lipid nanoparticles (LNPs) have garnered significant recognition as highly compatible and efficient delivery vehicles specifically tailored for mRNA, especially among active substances composed of nucleic acids. The remarkable clinical results achieved using LNPs have propelled them as promising candidates in the development of next-generation vaccines. LNPs possess a distinct advantage in their ability to facilitate the rapid design of mRNA and LNP platforms that can be seamlessly utilized interchangeably, even for mRNAs with distinct sequences. This streamlined approach has significantly compressed the development timeline compared to conventional vaccines, allowing for more expeditious responses to emerging infectious diseases and adaption to evolving viral strains.

Beyond their application in nucleic acids, LNPs exhibit remarkable versatility. By employing microfluidic processes, the precise encapsulation of proteins within LNPs becomes possible, greatly expanding their potential applications in advanced gene-editing technologies. This breakthrough capability opens up new avenues for targeted protein delivery and manipulation, broadening the scope of LNP-based therapeutic interventions. LNPs, with their inherent capability to encapsulate and deliver an extensive range of bioactive molecules, hold immense promise for the development of personalized medicine. Tailored therapies based on nucleic acids, proteins, or even a combination of both can be effectively and precisely delivered to target cells or tissues, providing a highly precise and patient-specific treatment approach.

In conclusion, LNPs have emerged as a cutting-edge platform revolutionizing the field of vaccine development through their unrivaled efficacy in mRNA delivery. Although current research predominantly focuses on mRNA, the potential for utilizing LNPs with other types of biopharmaceuticals should not be overlooked. The choice of biopharmaceuticals may depend on the heterogeneity of the neoantigen being targeted and the specific type of disease to which it is applicable. Integrating microfluidic processes into the LNP production pipeline facilitates the precise encapsulation of both nucleic acids and proteins, enabling significant advancements in gene-editing technologies. The remarkable versatility, efficiency, and potential for personalized medicine position LNPs as a highly promising tool in the realm of therapeutic interventions and precision medicine, paving the way for groundbreaking advancements in patient care.

## Figures and Tables

**Table 1:** Examples of immune-modulating lipid-based nanoparticles.

Category	Cargo	Disease	Mode of Action	LNP Type	Ref.
Immune cell activation	Heme-oxygenase-1-targeting siRNA	Metastatic melanoma	Heme oxygenase-1 silencing PD-L1 blockade	iLNP	[43]
	Amyloid-β (Aβ) peptide, rapamycin	Alzheimer’s Disease	Generation of Aβ-specific Th cells and Treg cells	Cationic liposomes	[44]
	E7, CD70, CD40L, TLR4 mRNA	Cervical cancer	Antigen-specific CD8 T cell response	iLNP	[45]
	CD47- and PD-L1-targeting siRNA	Glioblastoma multiforme	Simultaneous silencing of CD47 and PD-L1	Cationic liposomes	[46]
	Cyclic di-GMP	Lymphoma, breast cancer	STING pathway activation CTLA4 blockade	Cationic liposomes	[47]
	Cyclic GMP-AMP	Malignant pleural effusion in lung cancer	STING pathway activation PD-L1 blockade	Anionic liposomes	[48]
	Interferon-gamma-targeting siRNA	Inflammatory bowel disease	Interferon gamma silencing	iLNP	[49]
	mRNA-encoding human fibroblast growth factor 21, steroid prodrug	-	Anti-inflammatory response	iLNP	[31]
	Polo-like kinase 1 and CD45 siRNA	-	Leukocyte-selective targeting	iLNP	[30]
	mRNA-encoding CD19-targeting CAR bearing the CD3ζ and 4–1BB costimulatory domains	Leukemia	CD19-targeting CAR expression in T cells	iLNP	[50]
Vaccination	EVM158 mRNA	Mousepox	Antigen-specific CD8 T cell response	cLNP	[23]
	plasmid DNA encoding TGF-β single guide RNA and Cas9 protein	Melanoma	Transforming growth factor-β editing In situ vaccination of tumor-associated antigens	Cationic liposomes	[51]
	Self-amplifying RNA (saRNA) encoding the influenza hemagglutinin glycoprotein, SARS-CoV-2 spike protein	Influenza	Antigen-specific humoral and cellular response	iLNP	[25]
	mRNA encoding neoantigen	Lewis lung carcinoma	Antigen-specific cellular response	Cationic liposomes	[40]
	siRNA encoding the rabies virus glycoprotein	Rabies	Antigen-specific humoral and cellular response	cLNP	[33]

**Table 2:** FDA-approved lipid-based products.

Brand Name	Cargo	Indication	Year	Company
Spikevax	mRNA	COVID-19	2020	Moderna (Cambridge, MA, USA)
Comirnaty	mRNA	COVID-20	2020	Pfizer-BioNTech (New York, NY, USA)
ONPATTRO	siRNA	Hereditary transthyretin-mediated amyloidosis	2018	Alnylam (Cambridge, MA, USA)
VYXEOS	Cytarabine/ daunorubicin	Acute myeloid leukemia	2017	Jazz Pharmaceuticals (Dublin, Ireland)
Onivyde	Irinotecan	Metastatic pancreatic cancer	2015	Merrimack (North Andover, MA, USA)
Marqibo	Vincristine	Philadelphia chromosome-negative acute lymphoblastic leukemia	2012	Spectrum (Reno, NV, USA)
Definity	Perflutren	Ultrasound enhancement for patients with suboptimal echocardiograms	2001	Lantheus Medical Imaging (North Billerica, MA, USA)
Visudyne	Verteporfin	Predominantly classic subfoveal choroidal neovascularization in patients with age-related macular degeneration (AMD), pathologic myopia, presumed ocular histoplasmosis	2000	Xediton Pharmaceuticals (Mississauga, ON, Canada)
AmBisome	Amphotericin B	A variety of serious fungal infections	1997	Gilead Sciences (Foster City, CA, USA)
DaunoXome	Daunorubicin	First-line therapy against advanced Kaposi’s sarcoma associated with HIV	1996	Galen (Craigavon, UK)
Doxil	Doxorubicin	Ovarian cancer, AIDS-related Kaposi sarcoma, and multiple myeloma	1995	Janssen (Beerse, Belgium)
Diprivan	Propofol	A sedative–hypnotic agent	1989	Fresenius Kabi (vor der Höhe, Germany)

## Data Availability

No new data were created or analyzed in this study. Data sharing is not applicable to this article.
